# The intestinal microbiota modulates the visceral sensitivity involved in IBS induced by restraint combined with tail clustering

**DOI:** 10.3389/fcimb.2025.1549617

**Published:** 2025-02-20

**Authors:** Na Deng, Siqin Xie, Qin Liu, Huiyi Peng, Leyao Fang, Junxi Shen, Xiaoyuan Lin

**Affiliations:** ^1^ College of Traditional Chinese Medicine, Hunan University of Chinese Medicine, Changsha, Hunan, China; ^2^ Hunan Key Laboratory of Traditional Chinese Medicine Prescription and Syndromes Translational Medicine, Changsha, Hunan, China; ^3^ The First Hospital of Hunan University of Chinese Medicine, Changsha, Hunan, China

**Keywords:** irritable bowel syndrome (IBS), emotional stress, restraint, tail clipping, restraint combined with tail clipping, visceral sensitivity

## Abstract

**Objective:**

To compare three common stimuli that induce emotional stress to identify the optimal method for establishing an animal model that aligns with the clinical pathogenesis of irritable bowel syndrome (IBS) and to explore the gut microbiota mechanisms underlying IBS development.

**Methods:**

Thirty-six SPF-grade female Kunming mice were randomly divided into four groups: the normal control (NC) group, the restraint stress (BM) group, the tail clamp stress (CTM) group, and the restraint combined with tail clamp stress (BCTM) group, with 9 mice in each group. The NC group was fed normally without any stimulation. The BM group was subjected to restraint stress. The CTM group received intermittent tail clamp stress. The BCTM group underwent both restraint stress and intermittent tail clamp stress. The stimulation time for each group was 1 hour, and the modeling duration was 7 days. General behavioral changes in the mice were observed. The fecal water content was measured and calculated. The pain threshold, gastric residue rate, small intestine propulsion rate, and serum levels of short-chain fatty acids (SCFAs), serotonin (5-HT), interleukin-10 (IL-10), and tumor necrosis factor-alpha (TNF-α) were assessed. Histopathological analysis of the small intestine and colon tissues was performed. 16S rRNA high-throughput sequencing was subsequently conducted. The effects of different stimuli on mouse symptoms, gastrointestinal motility, visceral hypersensitivity, inflammation levels, and the gut microbiota were analyzed, and correlation analysis was performed.

**Results:**

Compared with the NC group, the BM, CTM, and BCTM groups of mice presented varying degrees of emotional hyperreactivity, accompanied by significantly reduced food intake and fecal water content and markedly elevated levels of inflammation, all of which are indicative of IBS symptoms. Among them, the BCTM group presented the most pronounced emotional hyperreactivity and irritability. The mice in the BCTM group had significantly higher gastric residue rates and 5-HT levels, with a marked reduction in pain tolerance. The gut microbiota of the mice in the BM, CTM, and BCTM groups all exhibited dysbiosis, with changes in the diversity, structural composition, and function of the microbial community. Specific bacterial taxa were enriched in each stress group, and their corresponding KEGG pathways were also significantly altered. Correlation analysis revealed that SCFAs were significantly positively correlated with the small intestine propulsion rate, whereas 5-HT was positively correlated with the gastric residue rate and negatively correlated with the pain threshold. SCFAs were positively correlated with IL-10 and TNF-α, and 5-HT was significantly positively correlated with IL-10 and TNF-α. In the BCTM group, the characteristic bacteria *Acinetobacter* and *Akkermansia* were significantly correlated with SCFAs and 5-HT.

**Conclusion:**

1. The restraint combined with the tail clamp stress method is superior among the three stress protocols and successfully induces the IBS mouse model. 2. *Acinetobacte*r and *Akkermansia* may contribute to the development of IBS induced by restraint combined with tail clamp stress through the regulation of SCFAs and 5-HT.

## 1 Introduction

**Figure 1:**
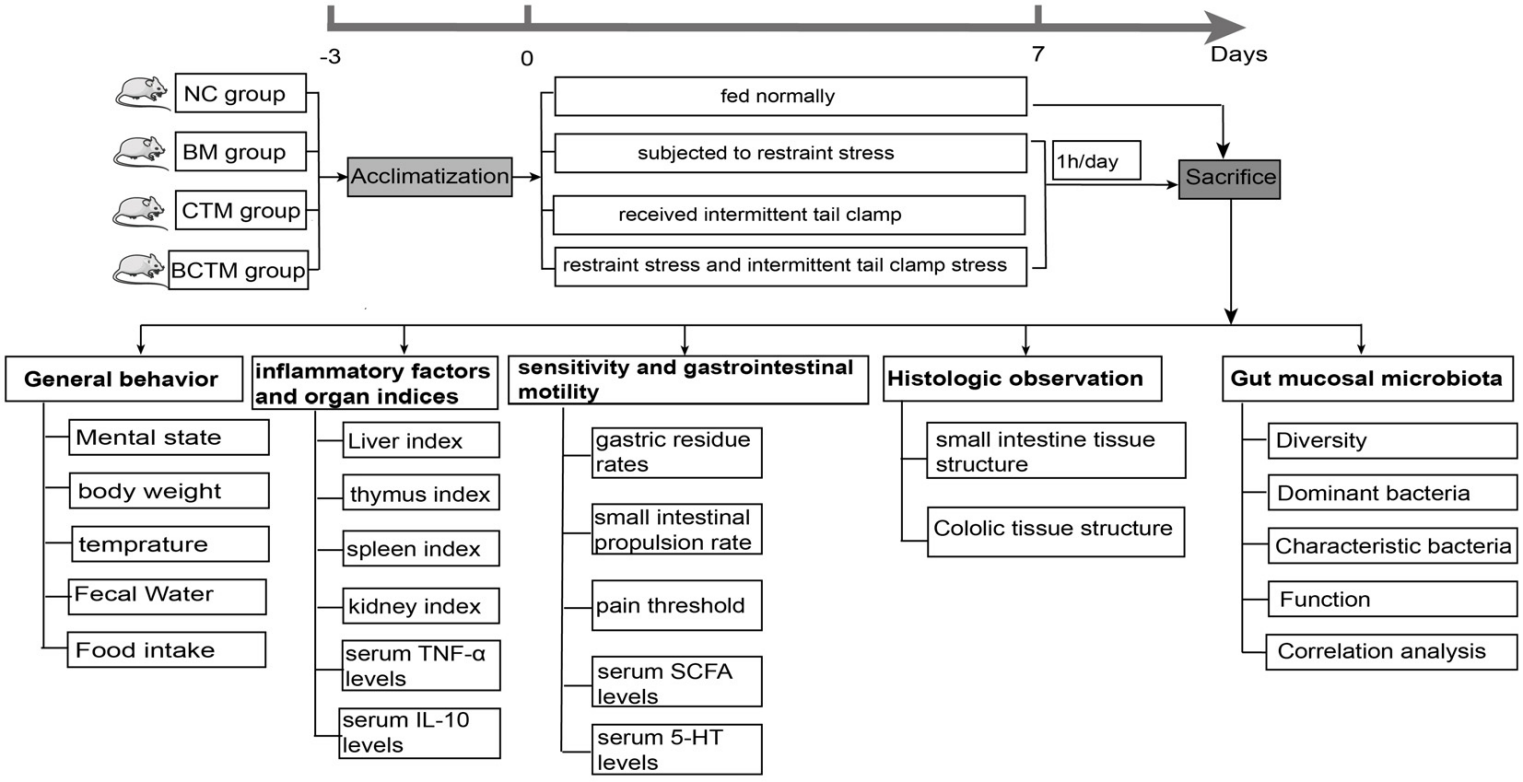
The experimental flow chart.

Irritable bowel syndrome (IBS) is a common functional gastrointestinal disorder characterized by recurrent abdominal pain, bloating, or discomfort and is often associated with abnormalities in bowel movements, such as altered stool form and frequency (Study group of functional gastrointestinal disorders et al., 2020). The global prevalence of IBS is 11.20%, with a noticeable increasing trend. In China, the prevalence varies widely, ranging from 5.67% to 13.10% (Liu et al., 2022). The etiology and pathogenesis of IBS remain unclear. The prevailing theory suggests that IBS is a biopsychosocial disease resulting from the interplay of multiple mechanisms and factors (Yang et al., 2023). The key pathological features of IBS include visceral hypersensitivity, abnormal gastrointestinal motility, brain−gut axis dysfunction, and gut microbiota dysbiosis. Furthermore, emerging evidence indicates a close association between gut microbiota imbalances and the onset of IBS (Gwee et al., 2019). However, the underlying mechanisms by which the gut microbiota contributes to the development of IBS remain unclear. IBS is characterized by a high incidence, difficult treatment, and, at present, no clear curative method. In the 2017 consensus, Zhang Shengsheng et al (Zhang et al., 2017). emphasized that IBS is often triggered by emotional factors, with spleen and stomach weakness and/or liver stagnation playing crucial roles in its pathogenesis. The 2024 updated consensus reiterates that liver stagnation and spleen deficiency are key pathogenic mechanisms in IBS, highlighting the importance of emotional factors in IBS development (Bian et al., 2024). Restraint, tail clamping, and a combination of both are commonly used methods to induce emotional stress and can be employed to create models of liver stagnation and spleen deficiency or depression (Liu et al., 2020; Shi et al., 2017; Yang et al., 2024) The 2021 IBS consensus (Li et al., 2021) suggests that both acute and chronic stress can trigger or exacerbate IBS symptoms. Therefore, in this study, we employed three different emotional stress-inducing stimuli—restraint, tail-clamping, and the combination of restraint and tail-clamping—to establish IBS mouse models. We analyzed the effects of these stimuli on symptoms, visceral hypersensitivity, gastrointestinal motility, inflammation levels, and the gut microbiota to explore the optimal IBS mouse model that best reflects the clinical etiology of the disease and to elucidate the microbiological mechanisms underlying the pathogenesis of IBS. The specific experimental procedure is depicted in **Figure 1**.

## 2 Materials and methods

### 2.1 Animals and housing conditions

A total of 36 SPF-grade female Kunming mice, Four weeks of age, each weighing 20 ± 2 g, were provided by Hunan Slex Experimental Co., Ltd. (Animal Quality Certificate No. ZS-202307240020). The mice were housed at the Animal Experiment Center of Hunan University of Chinese Medicine under barrier conditions. The room temperature was controlled at 23–25°C, with a relative humidity of 50–70%. A 12-hour light/dark cycle was maintained, and the animals had free access to food and water. This experiment was approved by the Animal Ethics Committee of Hunan University of Chinese Medicine (Ethical Approval No. LL20230032901).In clinical practice, the incidence of IBS is higher in females than in males, and gender can influence the gut microbiota (Wu et al., 2022). To eliminate the impact of gender on the gut microbiota, only female mice were used in this experiment.

### 2.2 Reagents

ELISA kits for short-chain fatty acids (SCFAs, batch no. JM11498M1), serotonin (5HT, batch no. JM02726M1), interleukin-10 (IL-10, batch no. JM02459M1), and tumor necrosis factor-alpha (TNF-α, batch no. JM02415M1) were purchased from Jiangsu Jingmei Biotech Co., Ltd.

### 2.3 Grouping and Model Establishment

#### 2.3.1 Animal grouping

After a 3-day acclimatization period, the 36 SPF-grade female Kunming mice were randomly assigned to 4 groups via a random number table: the normal control (NC) group, the restraint stress (BM) group, the tail clamp (CTM) group, and the restraint combined with tail clamp stress (BCTM) group, with 9 mice in each group. The NC group was maintained under normal feeding conditions without any stress exposure.

#### 2.3.2 Animal model establishment

The model groups were established on the basis of modified methods from previous studies (Yuan et al., 2020; Zhang et al., 2020) To avoid the influence of biological rhythms on the intestinal microbiota, all procedures and measurements were performed at the same time each day, starting at 9:00 AM, for a duration of 7 days.The restraint stress group was subjected to 1 hour of restraint using a 50 mL centrifuge tube with a small opening at the front. The mice were placed inside the tube, and foam blocks were used to fill the excess space to limit their movement while ensuring airflow to the head region through ventilation holes.The Tail Clamp group had their tails clamped with a steel clip at the distal third of the tail for 1 hour. To prevent ischemic necrosis of the tail, intermittent tail clamping (every 1–3 min) was applied, depending on the condition of the mice.The restraint combined with tail clamp stress was subjected to both restraint using the centrifuge tube and tail clamping at the same time. The durations of restraint and intermittent tail clamping were both 1 hour.

The general behavior of the mice was observed, and the fecal water content was measured. The food intake, body weight, and rectal temperature were recorded on days 1, 4, and 7 of the modeling process. After 7 days of modeling, the pain threshold was assessed by measuring the abdominal withdrawal reflex, and the gastric residue and intestinal propulsion rates were evaluated in the mice.

### 2.4 Observation indicators and methods

#### 2.4.1 Macroscopic indicators

On the basis of the clinical manifestations of IBS (Wang, 1993; Li et al., 2021) and the “2020 Expert Consensus on Irritable Bowel Syndrome in China” (Song et al., 2021), macroscopic diagnostic criteria for the mouse IBS model were established: changes in fecal characteristics or appearance, irritability, and aggressive behavior. During the experiment, general behavioral indicators of the mice, including emotional state, fur appearance and color, and fecal characteristics, were observed. Stress-induced irritability was assessed on the basis of the presence of irritability and aggressive behavior. Behavioral indicators such as food intake, body weight, and rectal temperature were used to evaluate the stress responses of each group of mice. Fecal characteristics were assessed by measuring the fecal water content. Daily food intake was monitored by checking the cages and water bottles before the experiment to ensure that no leakage occurred. The quantified feed was added at a fixed time each day, and the remaining food was measured after 24 hours. The average daily food intake (g) = (amount added - amount remaining)/number of mice in the group. On days 0, 4, and 7 of modeling, the body weights and rectal temperatures of the mice were measured at 10:00 AM. Before measurement, the mice were gently stimulated at the anus to induce defecation. One researcher held the mouse gently, while the other researcher inserted a thermometer lubricated with paraffin oil approximately 6 cm into the anus, ensuring a smooth and calm procedure. The thermometer was left for 3–5 minutes until the reading stabilized, and the temperature was recorded. Fresh fecal samples were collected at 3:00 PM from days 1-7 of modeling, weighed for their wet mass, dried at 110°C to a constant weight, and the dry weight was recorded. The fecal water content (%) was calculated as follows: fecal water content (%) = (wet weight - dry weight)/wet weight × 100%.

#### 2.4.2 Minimum pain threshold detection

The pain threshold was determined according to reference (Yu et al., 2021). Three mice were randomly selected from each group to measure the pain threshold, for a total of 12 mice. The selected mice were fasted (but not water deprived) for 18 hours. The animals were subsequently anesthetized with isoflurane (purchased from Shandong Antemu Animal Technology Co., Ltd.; batch number 2023052902). A 6F catheter coated with paraffin oil and equipped with an air sac (the catheter was connected to a 1 mL syringe for injecting water into the air sac) was inserted through the anus, with the distal end of the air sac positioned 1 cm from the anus and fixed at the base of the mouse’s tail. The mice were then placed in a custom-made transparent plastic cage (20 cm×6 cm×6 cm), which limited their movement to only forward and backward without allowing them to turn. After the mice completely regained consciousness, they were allowed to adapt to the environment for 15 minutes before the minimum amount of water injected that caused pain, defined as the pain threshold, was recorded. Pain tolerance was evaluated when strong muscle contractions in the dorsal or abdominal region of the mouse or when the abdomen lifted off the ground were observed (Al-Chaer et al., 2000). Each group of mice was subjected to three repeated tests, with each test lasting 30 seconds and with a 5-minute interval between tests.

#### 2.4.3 Measurement of the gastric residual rate and the small intestinal propulsion rate

Following the fasting period described in section 1.4.2, the 12 mice were fasted for an additional 24 hours. Each mouse was gavaged with 0.4 mL of semisolid nutritional paste containing 1% charcoal. After 20 min, the mice were euthanized by cervical dislocation. The pylorus, cardia, and ileocecal junction were ligated, and the mesentery was separated. The stomach and small intestine were removed and weighed along with their contents. The stomach was then cut open, the paste inside was washed off, and the stomach was dried on absorbent paper before its net weight was weighed. The length of the small intestine was measured, and the distance from the pylorus to the front edge of the black semisolid paste was recorded. The following formulas were used for calculation: gastric residual rate (%) = (total stomach weight - net stomach weight)/coal semisolid paste mass×100%; small intestinal propulsion rate (%) = (propulsion length/total small intestine length)×100%.

#### 2.4.4 Organ Index Measurement

The remaining 6 mice in each group were euthanized by cervical dislocation under sterile conditions. Blood was collected by enucleation, and the spleen, kidneys, liver, and thymus were harvested for organ index measurement. The connective tissue was removed from the organs, which were then placed on clean filter paper to absorb any surface fluid before the wet weight of each organ was determined. The organ index was calculated via the following formula: organ index (%) = (organ weight/mouse body weight) × 100%.

#### 2.4.5 HE staining for observation of morphological changes in mouse small intestinal and colon tissues

Mouse small intestine and colon tissue samples were collected and fixed in 4% paraformaldehyde solution. The tissues were then dehydrated in gradient ethanol, cleared in xylene, embedded in paraffin, and sectioned. After hematoxylin and eosin (HE) staining, pathological changes in the tissues were observed under an optical microscope (Fang et al., 2025).

#### 2.4.6 ELISA to measure changes in serum SCFA, 5HT, IL-10, and TNF-α levels

Blood samples collected in section 1.4.4 were centrifuged at 3000 rpm for 15 minutes at 4°C (centrifuge radius 5 cm), and the upper serum layer was harvested. The serum levels of SCFAs, 5HT, IL-10, and TNF-αwere measured according to the kit instructions. The concentration of the standard substance was plotted on the x-axis, and the OD value was plotted on the y-axis. A standard curve was generated from the linear regression of the concentration and OD value. The sample’s OD value was substituted into the equation, and the sample concentration was calculated and then multiplied by the dilution factor to obtain the actual sample concentration.

#### 2.4.7 16S rRNA next-generation sequencing for gut microbiota analysis

Refer to the literature to collect intestinal contents (Zhou et al., 2024).After rapidly euthanizing the mice using the cervical dislocation method. Under sterile conditions, the mice were dissected, and the contents of the small intestine were carefully collected using sterile forceps. The collected samples were placed into sterile centrifuge tubes and stored at -80°C for subsequent use. Total DNA from the collected mouse gut contents was extracted, PCR amplification was performed on the target region, and 16S rRNA next-generation sequencing was conducted (Qiao et al., 2024). The diversity and composition of the gut microbiota were analyzed, including diversity analysis, community structure analysis, differential abundance analysis, species correlation network analysis, microbiota gene function prediction, and microbiota phenotype prediction.

#### 2.4.8 Statistical analysis

Statistical analysis was performed via GraphPad Prism 10.1.2 and SPSS 24.0 software. The data are presented as the means ± standard deviations. For normally distributed data with equal variance, one-way analysis of variance (ANOVA) was used, followed by the least significant difference (LSD) *post hoc* test for group comparisons. For data that were either not normally distributed or had unequal variances, the nonparametric Kruskal−Wallis test was applied. *p<* 0.05 was considered statistically significant, and *p<* 0.01 was considered highly significant.

## 3 Results and analysis

### 3.1 Effects of different stimulation methods on mouse behavior

#### 3.1.1 Observation of the general state of the mice

The mice in the normal control (NC) group exhibited normal activity, with smooth and well-groomed fur and moderately dry and dark brown feces. On day 5 of modeling, the behavior of the mice in the BM and CTM groups was characterized by elevated forelimbs and assumed a defensive standing posture. The mice in the BCTM group were observed to engage in fighting behaviors, accompanied by squealing sounds. The feces from all three stimulation groups gradually became drier and harder. By day 7 of modeling, the mice in the three stimulation groups exhibited varying degrees of aggression, with increased fighting and climbing behaviors and dry, hard feces. Among these, the BCTM group presented the most prominent symptoms, including restless behavior, increased resistance during gavage, and other IBS-related irritability symptoms.

#### 3.1.2 Changes in food intake

**Figure 2:**
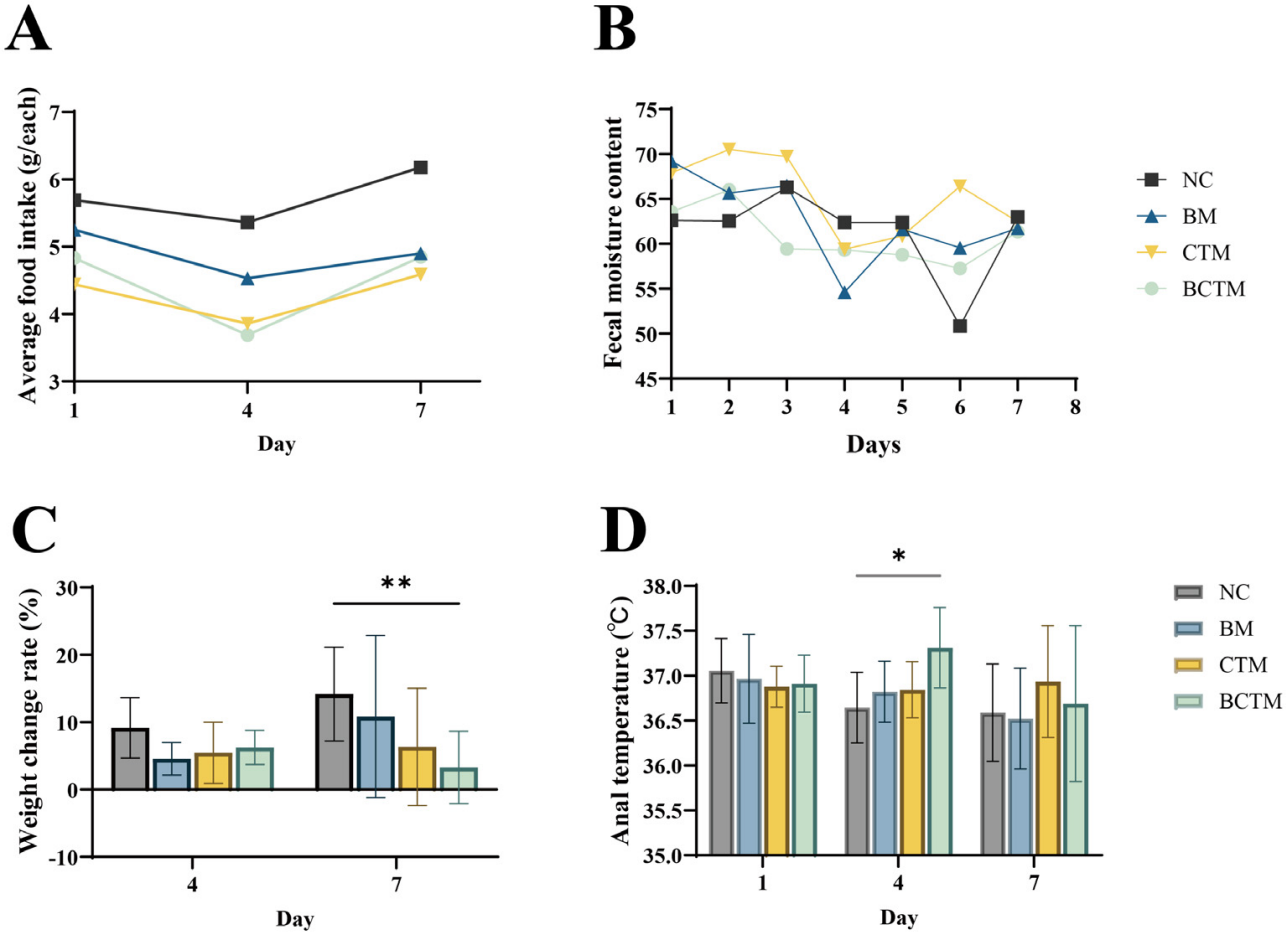
Effects of different stimuli on mouse behavior. **(A)** Food intake; **(B)** fecal water content; **(C)** body weight gain rate; **(D)** rectal temperature. The data are presented as the means ± standard deviations (n=9). *p<0.05 indicates a statistically significant difference, **p<0.01 indicates a highly significant difference. NC, normal control group; BM, restraint stimulation group; CTM, tail-clamping stimulation group; BCTM, restraint combined with tail-clamping stimulation group (subsequent figure legends are the same).

Compared with those in the NC group at the same time point, food intake in the BM, CTM, and BCTM groups was markedly lower, as shown in **Figure 2A** (food intake analysis: F=11.539, *p*=0.007).

#### 3.1.3 Changes in the body weight gain rate

Compared with those in the NC group at the same time point, on day 4 of modeling, the body weight gain rates in the BM, CTM, and BCTM groups tended to decrease (*p>*0.05). On day 7 of modeling, the body weight gain rate in the BM, CTM, and BCTM groups also decreased (*p>* 0.05; *p>* 0.05; *p<* 0.01), indicating that the weight gain rate in the BCTM group significantly decreased with increasing modeling duration, as shown in **Figure 2C**.

#### 3.1.4 Changes in rectal temperature

On the first day of modeling, the rectal temperature of the mice in all four groups stabilized at approximately 37°C. Compared with those in the NC group, on day 4 of modeling, the rectal temperatures in the BM, CTM, and BCTM groups tended to increase (*p>*0.05; *p>*0.05; *p<*0.01). On day 7 of modeling, the rectal temperature in the BM, CTM, and BCTM groups continued to increase (*p>*0.05; *p>*0.05; *p>*0.05), with only the BCTM group showing a significant increase in rectal temperature on day 4, as shown in **Figure 2D**.

#### 3.1.5 Changes in fecal water content

Fecal water content analysis revealed that, compared with that in the NC group, the fecal water content in the BM, CTM, and BCTM groups tended to decrease overall as the modeling time increased (F = 8.766, *p*= 0.006), as shown in **Figure 2B**.

### 3.2 Effects of different stimuli on visceral sensitivity and gastrointestinal motility

**Figure 3:**
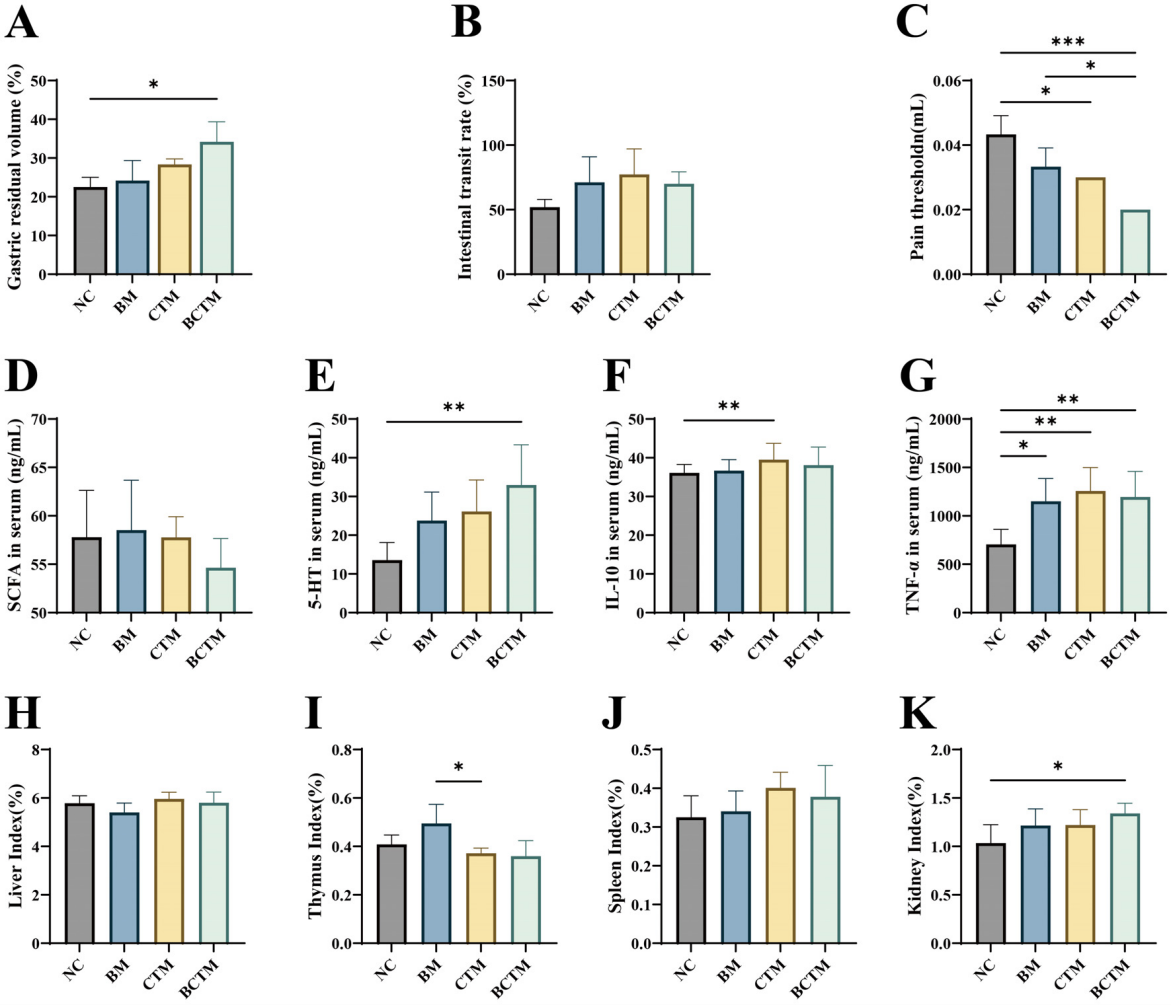
Effects of different stimuli on gastrointestinal motility, pain thresholds, serum biomarkers, and organ indices in mice. **(A)** Gastric residue rate; **(B)** small intestinal propulsion rate; **(C)** pain threshold; **(D)** serum SCFA levels; **(E)** serum 5-HT levels; **(F)** serum IL-10 levels; **(G)** serumTNF-α levels; **(H)** liver index; **(I)** thymus index; **(J)** spleen index; **(K)** kidney index. The data are presented as the means ± standard deviations (n=3/6). *p<0.05 indicates a significant difference, and **p<0.01 and ***p<0.001 indicate highly significant differences.

As shown in **Figures 3A, B**, compared with those in the NC group, the gastric residue rates in the BM, CTM, and BCTM groups were greater (*p>*0.05; *p>*0.05; *p<*0.05), whereas the small intestinal propulsion rate was also greater (*p>*0.05), with a significant increase in the gastric residue rate only in the BCTM group.

Compared with that in the NC group, the pain threshold was lower in the BM, CTM, and BCTM groups (*p>*0.05; *p<*0.05; *p<*0.01), as shown in **Figure 3C**.

As shown in **Figures 3D, E**, compared with those in the NC group, the serum SCFA levels were increased in the BM group but decreased in the CTM and BCTM groups, with no significant differences (*p>*0.05). The serum 5-HT levels were elevated in the BM, CTM, and BCTM groups (*p>* 0.05; *p>*0.05; *p<*0.01), with a significant increase in 5-HT only in the BCTM group.

### 3.3 Effects of different stimuli on inflammatory factors and organ indices in mice

As shown in **Figures 3F, G**, compared with those in the NC group, the serumTNF-α levels were significantly increased in the BM, CTM, and BCTM groups (*p<*0.05; *p<*0.01; *p<*0.01), and the serum IL-10 levels were also elevated (*p>*0.05; *p<*0.05; *p>*0.05).

As shown in **Figures 3H–K**, compared with those in the NC group, the liver organ indices were lower in the BM group, whereas the spleen, kidney, and thymus organ indices tended to increase (*p>* 0.05). In the CTM group, the thymus organ index tended to decrease, whereas the liver, spleen, and kidney organ indices tended to increase (*p>*0.05). In the BCTM group, the liver and thymus indices tended to decrease, whereas the spleen and kidney indices tended to increase (*p>*0.05; *p>*0.05; *p>* 0.05; *p<*0.05). Compared with that in the NC group, only the kidney index in the BCTM group was significantly greater.

### 3.4 Effects of different stimuli on colonic and small intestinal histopathology in mice

**Figure 4:**
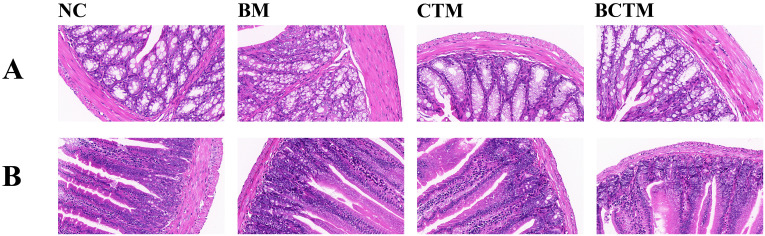
Effects of different stimuli on the histopathological morphology of mouse colon and small intestine tissues (HE, 400×). **(A)**. Histopathological morphology of colon tissue in each group of mice; **(B)**. Histopathological morphology of small intestine tissue in each group of mice.

As shown in **Figure 4A**, the colonic mucosa of the mice in the NC group was intact, with well-organized glands and no inflammatory cell infiltration. In the BM group, the colonic mucosa remained intact, but the glands were atrophied and disorganized, and there was a small amount of inflammatory cell infiltration and lymphoid tissue aggregation in the mucosal and submucosal layers. In the CTM and BCTM groups, the colonic mucosa was intact, with a relatively ordered glandular arrangement, accompanied by minor inflammatory cell infiltration.

**Figure 4B** shows that the overall structure of the small intestine in all groups of mice was normal, with well-preserved and orderly arrangement of the parenchymal structures. The villi length and density were normal, and the surface layer displayed a regular arrangement of columnar and goblet cells. The mucosal layer was intact without defects. In the BM, CTM, and BCTM groups, there was a slight accumulation of lymphocytes in the submucosal layer of the small intestine.

### 3.5 Effects of different stimuli on the gut microbiota in mice

#### 3.5.1 Quality assessment of gut microbiota sequencing in mice

**Figure 5:**
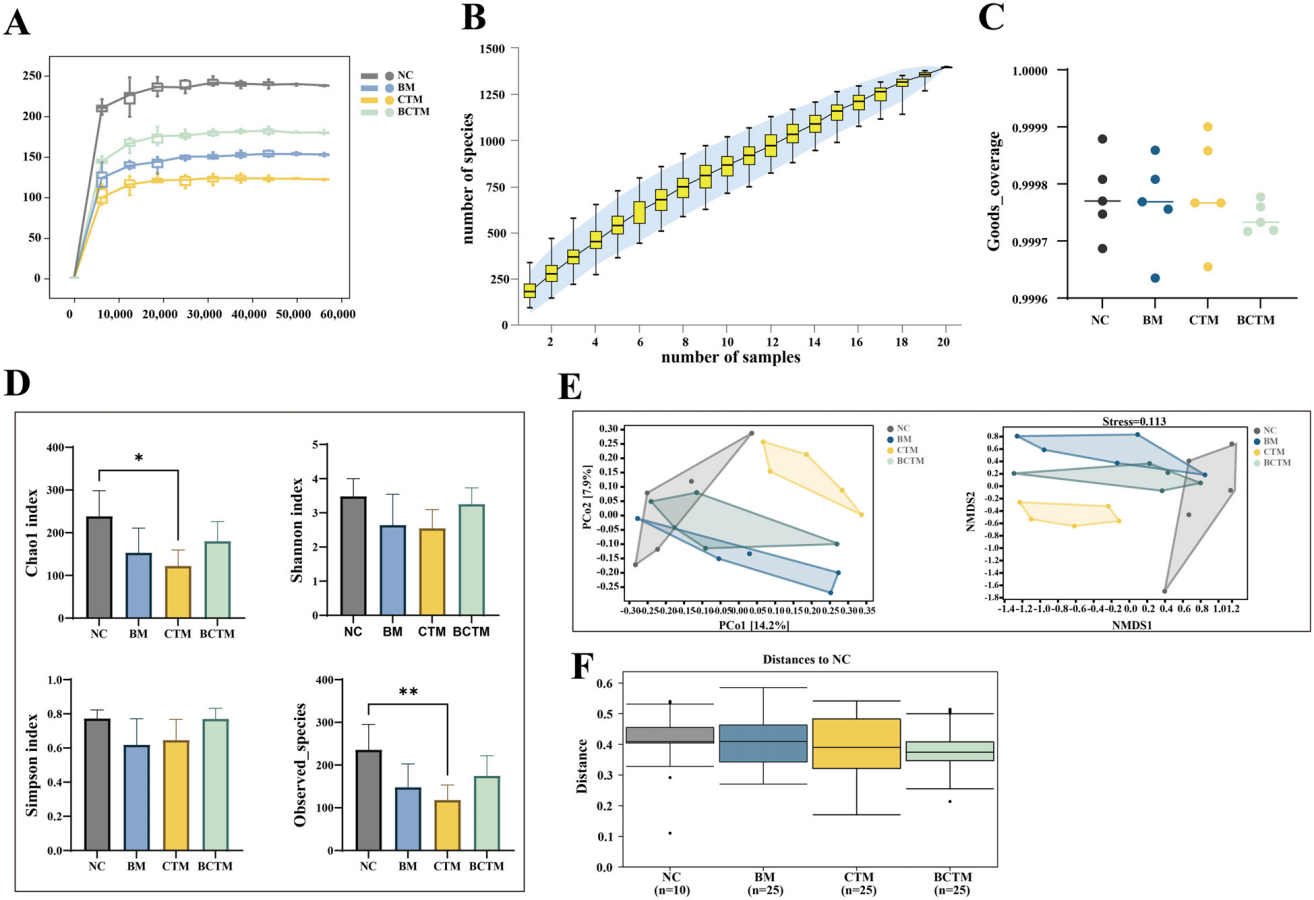
Quality assessment and diversity analysis of mouse gut contents via gut microbiota sequencing. **(A)** Chao1 dilution curve; **(B)** species accumulation curve; **(C)** Good’s coverage; **(D)** α diversity analysis; **(E)** PCoA and NMDS analysis; **(F)** intergroup differential analysis.

The sequencing depth was assessed by plotting the Chao1 rarefaction curve, which indirectly reflects the species richness in the samples. As shown in **Figure 5A**, the Chao1 rarefaction curves for all four groups flattened as the sequencing depth increased, indicating that the sequencing depth was sufficient for all four groups, covering most species and ensuring that species richness was adequate for subsequent analyses.

We performed random sampling and plotted species accumulation curves to assess the sufficiency of the sample size. As the sample size increased, the number of amplicon sequence variants (ASVs) increased more slowly and then plateaued, as shown in **Figure 5B**. This suggests that the sampling was sufficient for the needs of this study.

We also calculated the Good’s coverage index to evaluate the coverage of the samples within each group. The Good’s coverage index for all samples within each group was consistently above 99.96%, with no significant outliers, as shown in **Figure 5C**. This indicates that the sample coverage within each group was good and that the homogeneity of the samples met the experimental design requirements.

#### 3.5.2 Changes in the gut microbiota diversity and community structure of mice

Alpha diversity reflects the richness and diversity of species within individual samples. We used the Chao1 and Observed_species indices to evaluate species richness, and the Shannon and Simpson indices to assess species diversity. Compared to the NC group, the Chao1, observed species, Shannon, and Simpson indices were all lower in the BM and BCTM groups, although the differences were not significant (*p>*0.05). In the CTM group, both the Chao1 and Observed_species indices were significantly lower (*p<*0.05; *p<*0.01), whereas the Shannon and Simpson indices tended to decrease (*p>*0.05). These findings suggest that all three stimuli altered the alpha diversity of the gut microbiota in the small intestine of mice, leading to decreased richness and diversity. Among these, the tail-clamp stimulus caused a significant reduction in microbiota diversity (**Figure 5D**).

Beta diversity reflects the differences in species composition between samples. In this study, we used the Jaccard distance algorithm. As shown in **Figure 5E**, in the PCoA, the contribution of the first principal coordinate (PCo1) was 14.2%, and the second principal coordinate (PCo2) contributed 17.9%. The confidence intervals for the BM, CTM, and BCTM groups were clearly separated from those of the NC group, indicating good separation between the groups. The NMDS analysis revealed that the four groups of mice presented distinct community structure distributions, with a stress value of 0.113 (a stress value less than 0.2 indicates reliable results, and the lower the value is, the better). Intergroup analysis revealed that the within-group differences were smaller than the between-group differences (*p<*0.05), indicating that all three emotional stimuli significantly altered the gut microbiota community structure of the small intestine in mice (**Figure 5F**).

#### 3.5.3 Analysis of ASVs in the gut microbiota of mouse intestinal contents

**Figure 6:**
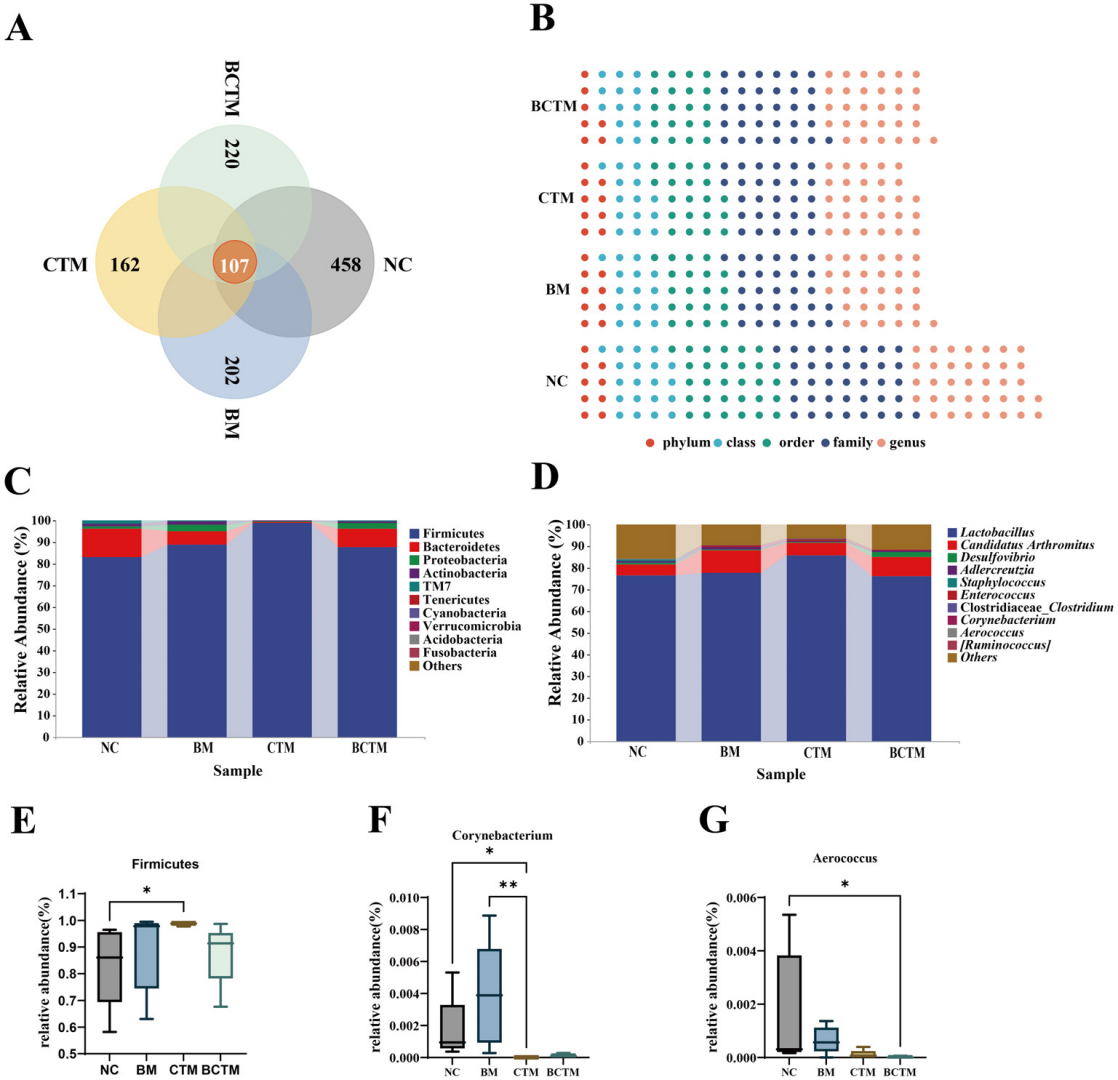
Changes in the microbial community composition of mouse gut contents. **(A)** Venn diagram; **(B)** Waffle chart; **(C)** Relative abundance at the phylum level; **(D)** Relative abundance at the genus level. **(E)** Dominant phylum (Firmicutes) with significant changes; **(F)** Dominant genus (Corynebacterium) with significant changes; **(G)** Dominant genus (Aerococcus) with significant changes.

The ASVs from the samples of each group were compared, and 107 common ASVs were found across the four groups of mouse small intestinal content samples. The numbers of ASVs in the NC, BM, CTM, and BCTM groups were 565, 309, 269, and 327, respectively. The number of ASVs in the BM, CTM, and BCTM groups was lower than that in the NC group, indicating that all three stimulation methods could alter the microbiota composition in the mouse small intestinal contents, resulting in a reduction in the number of ASVs, as shown in **Figure 6A**.

#### 3.5.4 Taxonomic Composition Analysis at Various Classification Levels

The changes in the taxonomic composition at different classification levels after different stimuli were presented were visualized via a pie chart (**Figure 6B**). The results revealed that, among the four groups, the NC group had the highest proportion of microbiota at each classification level. All three types of stimulation significantly altered the composition of the microbiota in the mouse intestinal contents.

#### 3.5.5 Analysis of dominant microbial groups in mouse intestinal contents

**Figure 6C** shows the relative abundance of the top 10 bacteria at the phylum level. The dominant phyla in the four groups included Firmicutes, Bacteroidetes, Proteobacteria, Actinobacteria, and TM7, which had relatively high proportions. Statistical analysis of these dominant phyla revealed that, compared with that in the NC group, the relative abundance of Firmicutes in the CTM group was significantly greater (83.18% vs 98.88%), As shown in **Figure 6E**. **Figure 6D** shows the relative abundance of the top 10 bacteria at the genus level. The analysis of the top 10 dominant genera revealed that, compared with that in the NC group, the relative abundance of *Corynebacterium* in the CTM group was significantly lower (0.1732% vs 0.0008%), as shown in **Figure 6F**, whereas in the BCTM group, the relative abundances of *Aerococcus* were extremely significantly lower, respectively (0.1674% vs 0.0019%), as shown in **Figure 6G**. These results indicate that tail clipping significantly altered the composition of the dominant microbial communities at both the phylum and genus levels in the mouse intestinal contents and that the combination of tail clipping with restraint stimulation had a significant effect on the genus-level microbiota composition in the mouse intestinal contents.

#### 3.5.6 Analysis of signature bacteria in mouse intestinal contents

**Figure 7:**
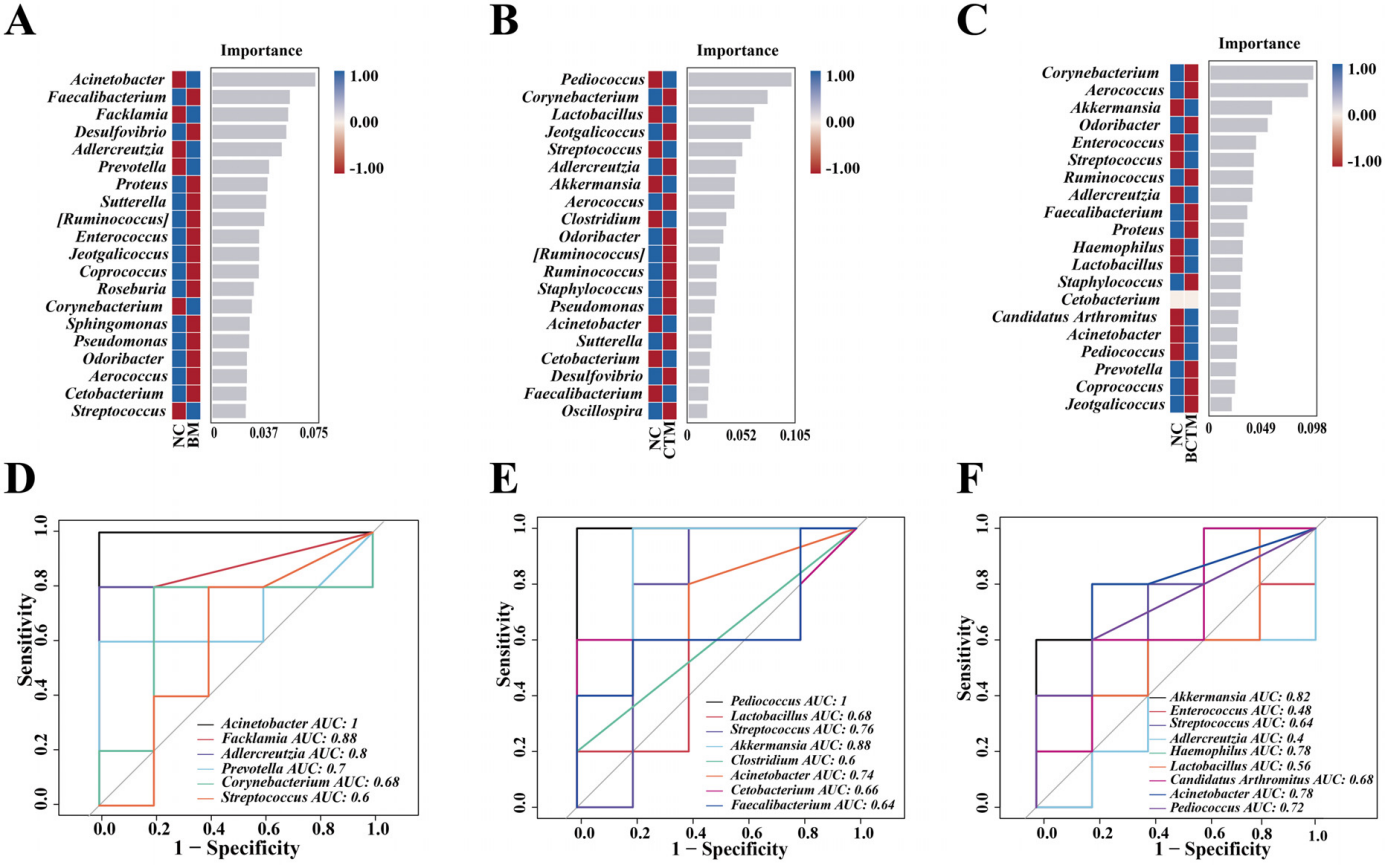
Analysis of the characteristic microbiota in mouse gut contents. **(A)** Random forest plot at the genus level for the BM group; **(B)** random forest plot at the genus level for the CTM group; **(C)** random forest plot at the genus level for the BCTM group; **(D)** ROC curve at the genus level for the BM group; **(E)** ROC curve at the genus level for the CTM group; **(F)** ROC curve at the genus level for the BCTM group.

**Figures 7A–C** show the signature bacterial genera at the genus level in the BM, CTM, and BCTM groups, respectively. We performed ROC analysis on the random forest model at the genus level for each group (**Figures 7D–F**). In the BM group, the signature genera *Acinetobacter* (AUC = 1), *Facklamia* (AUC = 0.88), and *Adlercreutzia* (AUC = 0.80) were identified. In the CTM group, the signature genera *Pediococcus* (AUC = 1) and *Akkermansia* (AUC = 0.88) were prominent. In the BCTM group, the signature genera *Akkermansia* (AUC = 0.82) and *Acinetobacter* (AUC = 0.78) presented relatively high AUC values. These results suggest that the above signature genera may serve as potential biomarkers for restraint, tail clipping, and the combination of restraint and tail clipping stimulation.

#### 2.5.7 Changes in gut microbiota function in mice

**Figure 8:**
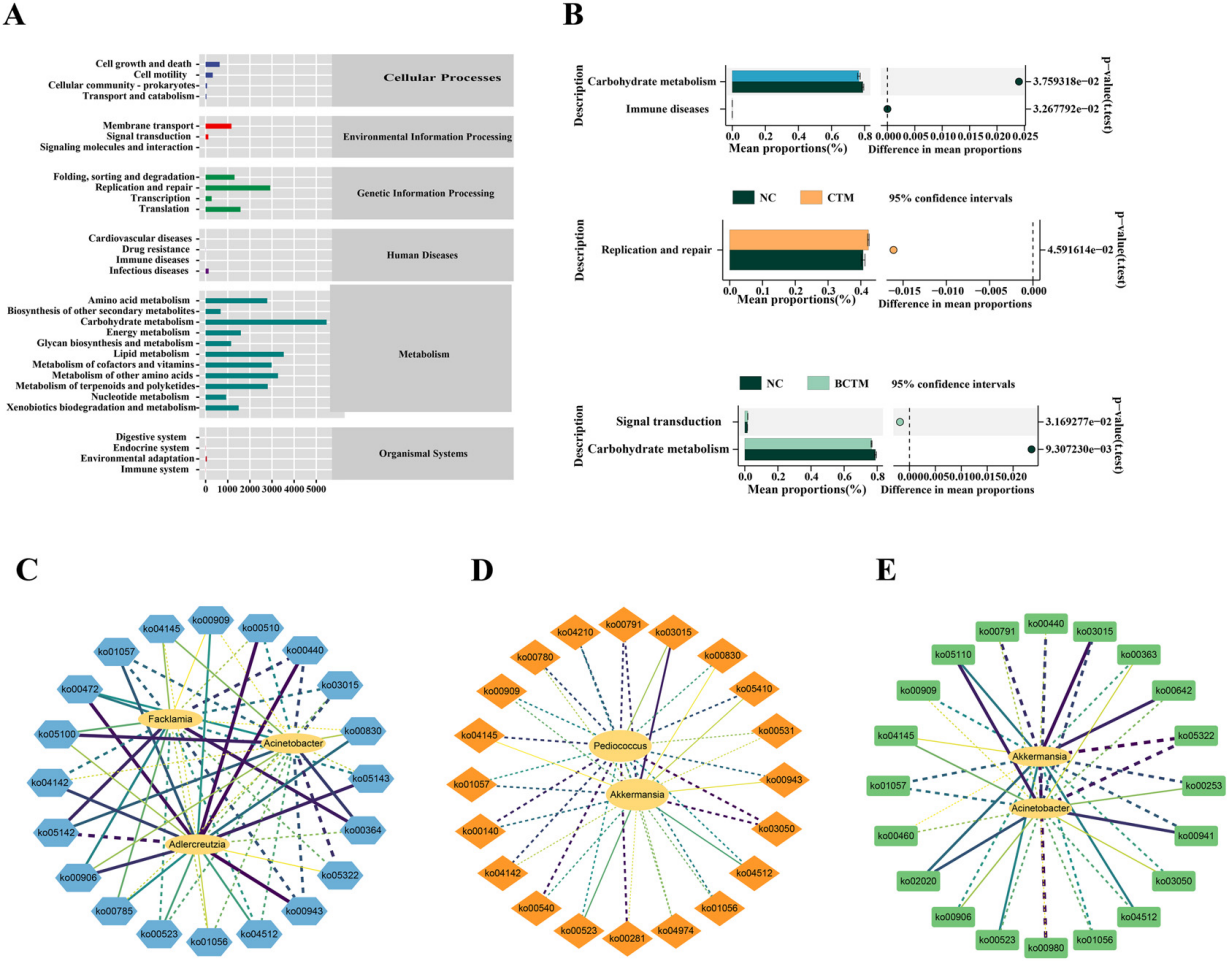
Effects of different stimuli on mouse KEGG pathways. **(A)** KEGG functional prediction abundance plot; **(B)** significantly different KEGG secondary functions between groups; **(C)** network plot of characteristic genera and KEGG pathways for the BM group; **(D)** network plot of characteristic genera and KEGG pathways for the CTM group; **(E)** network plot of characteristic genera and KEGG pathways for the BCTM group. The solid lines represent positive correlations, the dashed lines represent negative correlations, and the thicker and darker the line is, the more significant the correlation.

As shown in **Figure 8A**, KEGG functional clustering analysis revealed that the first-level functions of the gut microbiota in the intestinal contents were classified into six categories, and the second-level functions were further divided into 30 subcategories. Among them, the microbiota related to metabolic functions accounted for the largest proportion. As shown in **Figure 8B**, in comparison with the NC group, the BM group presented a significant reduction in the relative abundance of carbohydrate metabolism and immune disease functional microbiota; the CTM group presented a significant increase in the relative abundance of replication and repair functional microbiota; and the BCTM group presented a significant increase in the relative abundance of signal transduction functional microbiota and a significant decrease in carbohydrate metabolism functional microbiota.

On the basis of the PICRUSt2 analysis results of functional potential prediction from the KEGG database and the KEGG pathway differential analysis, we screened the top 20 KEGG pathways with significant differences between the three stimulation groups and the normal group. A correlation analysis was then performed between these pathways and the signature bacteria. The “signature bacteria genus-KEGG pathway” interaction networks for the BM, CTM, and BCTM groups were constructed via Cytoscape 3.9.1 (**Figures 8C–E**). The results of the correlation analysis revealed that in the BM group, the signature bacterial genus *Adlercreutzia* was significantly negatively correlated with Chagas disease American trypanosomiasis (*p<*0.05) and significantly positively correlated with D-arginine and D-ornithine metabolism, N-glycan biosynthesis, phosphonate and phosphinate metabolism, and isoflavonoid biosynthesis (*p<*0.05; *p<*0.01; *p<*0.01; *p<*0.01). *Facklamia* was significantly positively correlated with fluorobenzoate degradation (*p<*0.05). In the CTM group, *Pediococcus* was significantly negatively correlated with proteasome and geraniol degradation (*p<* 0.05; *p<*0.05), and *Akkermansia* was significantly negatively correlated with proteasome activity (*p<* 0.05). In the BCTM group, *Akkermansia* was significantly negatively correlated with the metabolism of xenobiotics by cytochrome P450 and SLE (*p<*0.05; *p<*0.05).These findings suggest that the aforementioned KEGG pathways may represent key pathways through which the three different stimuli influence changes in the gut microbiota of mice.

### 3.6 Correlation analysis

#### 3.6.1 Correlation analysis between visceral hypersensitivity-related indicators and gastrointestinal motility, pain tolerance, and inflammatory indicators in mice

**Figure 9:**
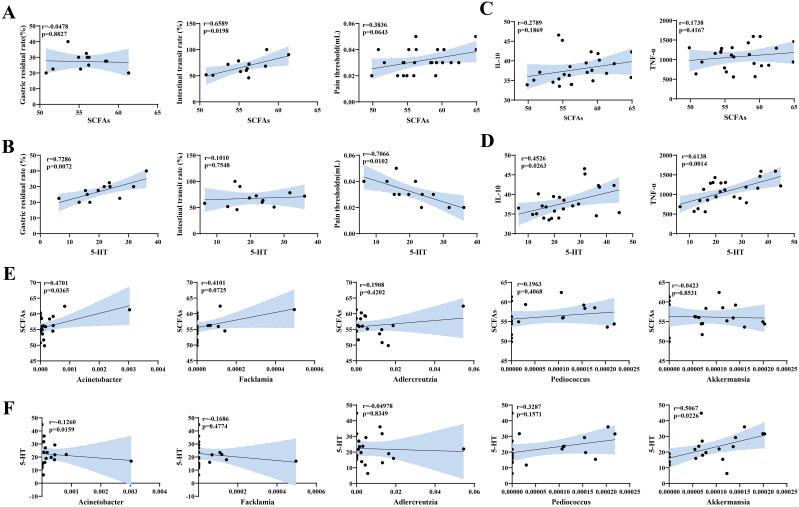
Correlation analysis between visceral hypersensitivity-related indices, gastrointestinal motility, pain thresholds, inflammation-related indices, and characteristic microbiota in mouse gut contents. **(A)** Scatter plot of SCFA levels and gastrointestinal motility and pain threshold indices. **(B)** Scatter plot of 5-HT levels and gastrointestinal motility and pain threshold indices. **(C)** Scatter plot of SCFA and inflammation indices. **(D)** Scatter plot of 5-HT and inflammation indices. **(E)** Scatter plot of characteristic genera and SCFAs. **(F)** Scatter plot of characteristic genera and 5-HT.

We analyzed the correlations between visceral hypersensitivity-related indicators (SCFAs and 5-HT) and gastrointestinal motility and pain tolerance indicators (gastric retention rate, intestinal propulsion rate, and pain threshold), and the results are presented in scatter plots. SCFAs were negatively correlated with the gastric retention rate, positively correlated with the pain threshold, with no significant correlations, and significantly positively correlated with the intestinal propulsion rate (*p<*0.05) (**Figure 9A**). 5-HT was significantly positively correlated with the gastric retention rate (*p<*0.01), significantly negatively correlated with the pain threshold (*p<*0.05), and positively correlated with the intestinal propulsion rate (*p>*0.05) (**Figure 9B**).

Additionally, we analyzed the correlations between visceral hypersensitivity-related indicators (SCFAs, 5-HT) and inflammatory indicators (IL-10,TNF-α). The results revealed that SCFAs were positively correlated with both IL-10 andTNF-α, but the correlations were not significant (**Figure 9C**). In contrast, 5-HT was significantly positively correlated with IL-10 (*p<*0.05) andTNF-α (*p<*0.01) (**Figure 9D**).

#### 3.6.2 Correlation analysis between signature bacteria and visceral hypersensitivity-related indicators

Next, we analyzed the correlations between the signature bacteria *Acinetobacter*, *Facklamia*, *Adlercreutzia*, *Pediococcus*, and *Akkermansia* and visceral sensitivity indicators (SCFAs and 5-HT). The results are shown in **Figures 9E, F**. *Facklamia* and *Adlercreutzia* were positively correlated with SCFAs and negatively correlated with 5-HT. *Pediococcus* was positively correlated with both SCFAs and 5-HT. *Akkermansia* was negatively correlated with SCFAs, but the correlations were not significant. Only *Acinetobacter* was significantly positively correlated with SCFAs (*p<*0.05) and significantly negatively correlated with 5-HT (*p<*0.05), whereas *Akkermansia* was significantly positively correlated with 5-HT (*p<*0.05).

## 4 Discussion

### 4.1 Evaluation analysis of the IBS model

Restraint, tail clamping, and the combination of restraint with tail clamping are stress-inducing methods that limit the activity of mice and induce painful stimuli, leading to negative emotional stress, such as tension, anxiety, fear, and irritability. This, in turn, results in liver Qi overacting on the spleen, causing liver stagnation and spleen deficiency (Liu et al., 2020), which is considered a key pathogenesis for irritable bowel syndrome (IBS). The modeling methods involving emotional stressors are consistent with clinical scenarios where stress from work, study, and other sources leads to negative emotional states such as tension, anxiety, and depression, which can trigger the onset and development of IBS. Gastrointestinal motility disorders and visceral hypersensitivity are key factors in IBS pathogenesis (Gwee et al., 2019). The pain threshold can reflect visceral sensitivity: the lower the pain threshold is, the poorer the body’s tolerance to pain and the greater the visceral sensitivity (Al-Chaer et al., 2000). The fecal water content was used to measure the fecal characteristics of the mice in the different groups. SCFAs, as important metabolites of the gut microbiota, have various functions, including regulating gut immunity, exerting anti-inflammatory effects, and protecting the intestinal mucosal barrier (Yao et al., 2022). Their levels tend to increase in IBS-D patients (diarrhea-predominant IBS) and decrease in IBS-C patients (constipation-predominant IBS) (Ge et al., 2018). Studies have suggested that serotonin (5-HT) might influence visceral sensitivity by regulating gastrointestinal secretion and motility (Asano et al., 2017), whereas SCFAs may increase intestinal 5-HT levels, inducing visceral hypersensitivity and altering fecal characteristics in IBS-D patients (Shaidullov et al., 2021) (Long et al., 2018). A literature review by Hang et al. (Hang et al., 2021) revealed that probiotics can alleviate abdominal pain and diarrhea symptoms in IBS-D patients by increasing SCFA levels in the gut. IL-10 is a key anti-inflammatory cytokine that limits immune cell activation and cytokine production in innate immune cells (Saraiva and O'Garra, 2010).TNF-α is a cytokine produced by immune cells under inflammatory or infectious stimuli that triggers inflammation while also participating in regulating immune and inflammatory responses (Wen et al., 2024). Previous studies have shown that SCFAs can activate the nuclear factor kappa-B (NF-κB) pathway in intestinal epithelial cells, increasingTNF-α and reducing IL-10 secretion (Bolte et al., 2021), whereasTNF-α can affect the synthesis, release, and reuptake of 5-HT (Shelton and Miller, 2011). Thus, SCFAs and 5-HT may influence visceral hypersensitivity by regulating gastrointestinal motility and the secretion of inflammatory factors.Compared with the NC group, the BM, CTM, and BCTM groups presented varying degrees of emotional irritability, accompanied by reduced food intake, altered fecal characteristics, lower fecal water content, and elevated inflammation levels, which are characteristic symptoms of IBS, seven days after modeling. However, there was no significant damage to the small intestine or colon tissue structure, which aligns with the nondamaging inflammatory nature of IBS. Among these, the BCTM group presented the most prominent emotional irritability and hypersensitivity symptoms. Additionally, the BCTM group presented significantly increased gastric retention rates and 5-HT levels, along with a marked decrease in pain tolerance. These findings are consistent with gastrointestinal motility disorders and increased visceral sensitivity in IBS patients, suggesting that the combination of restraint and tail clamping is the most effective method among the three stressors tested and successfully establishes an IBS mouse model.

### 4.2 Relationships between visceral hypersensitivity induced by restraint and tail clamping stimulation and gut microbiota dysbiosis in IBS

Multiple studies support the significant role of gut microbiota dysbiosis in the pathogenesis of IBS (Ghoshal and Srivastava, 2014; Ghoshal et al., 2016; Pimentel et al., 2011). Further research has shown that the gut microbiota and its metabolic products interact with both the enteric and central nervous systems, playing a key role in IBS (Gracie et al., 2019). *Acinetobacter* species, including both opportunistic pathogens and harmless saprophytic bacteria, have drawn attention because of concerns over antibiotic resistance (Lucidi et al., 2019). These species exhibit virulence characteristics that allow them to evade rapid clearance by the innate immune system (Wong et al., 2017). However, there is limited research on the involvement of *Acinetobacter* in the regulation of visceral hypersensitivity. On the other hand, *Akkermansia*, a well-known probiotic, has been reported to alter the gut bacterial composition and short-chain fatty acid (SCFA) production and is involved in the development of mental disorders. Additionally, *Akkermansia* has immunoregulatory and anti-inflammatory properties and can increase serotonin (5-HT) levels to alleviate depression (Cheng et al., 2021; Fujisaka et al., 2020; Gordon and Goelman, 2016; Xia et al., 2022). Zhang Mengmeng et al (Zhang et al., 2023)summarized the literature and suggested that an imbalance in 5-HT synthesis and transport may trigger the onset and development of IBS.The present study revealed that three different types of stimuli significantly altered the diversity and community structure of the gut microbiota in the small intestine of mice. Moreover, different characteristic bacterial genera were enriched in different groups. *Acinetobacter*, *Facklamia*, and *Adlercreutzia* were significantly enriched in the BM (restraint and tail clamping) group; *Pediococcus* and *Akkermansia* were notably enriched in the CTM (control, tail clamping) group; and both *Akkermansia* and *Acinetobacter* were notably enriched in the BCTM (restraint combined with tail clamping) group. We hypothesize that these characteristic genera may serve as potential biomarkers for restraint, tail clamping, and combined restraint–tail clamping stimulation. Correlation analysis results showed that SCFAs were significantly positively correlated with intestinal propulsion rate; 5-HT was significantly positively correlated with gastric residue rate and significantly negatively correlated with pain threshold. Additionally, SCFAs were positively correlated with IL-10 and TNF-α, while 5-HT was significantly positively correlated with IL-10 and TNF-α. Previous literature suggests that SCFAs and 5-HT may influence visceral hypersensitivity by regulating gastrointestinal motility and the secretion of inflammatory factors. Therefore, SCFAs and 5-HT may play a positive regulatory role in gastric residue and intestinal propulsion rates, a negative regulatory role in pain threshold, and exhibit a positive regulatory effect on inflammatory factors IL-10 and TNF-α.Furthermore the characteristic bacterium *Acinetobacter* was significantly positively correlated with SCFAs and significantly negatively correlated with 5-HT, whereas *Akkermansia* was positively correlated with 5-HT. These findings suggest a regulatory interaction between *Acinetobacter* and SCFAs, 5-HT, and between *Akkermansia* and 5-HT. However, the underlying biological functions and mechanisms of these interactions require further investigation.

In summary, different stimuli have varying effects on mouse behavior, gastrointestinal motility, pain tolerance, visceral hypersensitivity, inflammatory markers, and the structure and function of the gut microbiota. Among the three types of stimuli, restraint combined with tail clamping (BCTM) demonstrated superiority and successfully induced the IBS model. Restraint plus tail clamping resulted in increased visceral hypersensitivity markers in mice, along with gastrointestinal motility disturbances, reduced pain tolerance, and elevated levels of inflammation. Moreover, the visceral hypersensitivity indicators (SCFAs, 5-HT) were positively correlated with gastric residue and intestinal propulsion rates, negatively correlated with pain threshold, and positively correlated with elevated levels of body inflammation. Therefore, the combined restraint and tail-clamp stimulation may accelerate gastrointestinal motility and increase inflammation, leading to the development of IBS by affecting visceral hypersensitivity. Akkermansia and Acinetobacter are characteristic microbiota in the combined restraint and tail-clamp group, thus closely related to the occurrence of IBS induced by this stimulus. Moreover, Akkermansia and Acinetobacter have significant regulatory effects on SCFAs and 5-HT. Therefore, We speculate that Acinetobacter and Akkermansia may contribute to the development of IBS induced by combined restraint and tail-clamp by regulating SCFAs and 5-HT. SCFAs and 5-HT may interfere with the gut microbiota imbalance, gastrointestinal motility disorders, reduced pain tolerance, and elevated inflammation levels caused by the combined restraint and tail-clamp.

## Data Availability

The data presented in the study are deposited in the NCBI repository, accession number http://www.ncbi.nlm.nih.gov/bioproject/1210116.
